# Surveillance Colonoscopy Findings in Older Adults With a History of Colorectal Adenomas

**DOI:** 10.1001/jamanetworkopen.2024.4611

**Published:** 2024-04-02

**Authors:** Jeffrey K. Lee, Abhik Roy, Christopher D. Jensen, Jennifer T. Chan, Wei K. Zhao, Theodore R. Levin, Jessica Chubak, Ethan A. Halm, Celette S. Skinner, Joanne E. Schottinger, Nirupa R. Ghai, Andrea N. Burnett-Hartman, Aruna Kamineni, Natalia Udaltsova, Douglas A. Corley

**Affiliations:** 1 Division of Research, Kaiser Permanente Northern California, Oakland; 2 Kaiser Permanente San Leandro Medical Center, San Leandro, California; 3 Kaiser Permanente Washington Health Research Institute, Seattle; 4 Kaiser Permanente Bernard J. Tyson School of Medicine, Pasadena, California; 5 Rutgers Biological Health Sciences, Rutgers University, New Brunswick, New Jersey; 6 Department of Internal Medicine, University of Texas Southwestern Medical Center, Dallas; 7 Simmons Comprehensive Cancer Center, University of Texas Southwestern Medical Center, Dallas; 8 Peter O’Donnell Jr School of Public Health, University of Texas Southwestern Medical Center, Dallas; 9 Kaiser Permanente Southern California Department of Research and Evaluation, Pasadena; 10 Department of Quality and Systems of Care, Kaiser Permanente Southern California, Pasadena; 11 Kaiser Permanente Colorado Institute for Health Research, Aurora

## Abstract

**Importance:**

Postpolypectomy surveillance is a common colonoscopy indication in older adults; however, guidelines provide little direction on when to stop surveillance in this population.

**Objective:**

To estimate surveillance colonoscopy yields in older adults.

**Design, Setting, and Participants:**

This population-based cross-sectional study included individuals 70 to 85 years of age who received surveillance colonoscopy at a large, community-based US health care system between January 1, 2017, and December 31, 2019; had an adenoma detected 12 or more months previously; and had at least 1 year of health plan enrollment before surveillance. Individuals were excluded due to prior colorectal cancer (CRC), hereditary CRC syndrome, inflammatory bowel disease, or prior colectomy or if the surveillance colonoscopy had an inadequate bowel preparation or was incomplete. Data were analyzed from September 1, 2022, to February 22, 2024.

**Exposures:**

Age (70-74, 75-79, or 80-85 years) at surveillance colonoscopy and prior adenoma finding (ie, advanced adenoma vs nonadvanced adenoma).

**Main Outcomes and Measures:**

The main outcomes were yields of CRC, advanced adenoma, and advanced neoplasia overall (all ages) by age group and by both age group and prior adenoma finding. Multivariable logistic regression was used to identify factors associated with advanced neoplasia detection at surveillance.

**Results:**

Of 9740 surveillance colonoscopies among 9601 patients, 5895 (60.5%) were in men, and 5738 (58.9%), 3225 (33.1%), and 777 (8.0%) were performed in those aged 70-74, 75-79, and 80-85 years, respectively. Overall, CRC yields were found in 28 procedures (0.3%), advanced adenoma in 1141 (11.7%), and advanced neoplasia in 1169 (12.0%); yields did not differ significantly across age groups. Overall, CRC yields were higher for colonoscopies among patients with a prior advanced adenoma vs nonadvanced adenoma (12 of 2305 [0.5%] vs 16 of 7435 [0.2%]; *P* = .02), and the same was observed for advanced neoplasia (380 of 2305 [16.5%] vs 789 of 7435 [10.6%]; *P* < .001). Factors associated with advanced neoplasia at surveillance were prior advanced adenoma (adjusted odds ratio [AOR], 1.65; 95% CI, 1.44-1.88), body mass index of 30 or greater vs less than 25 (AOR, 1.21; 95% CI, 1.03-1.44), and having ever smoked tobacco (AOR, 1.14; 95% CI, 1.01-1.30). Asian or Pacific Islander race was inversely associated with advanced neoplasia (AOR, 0.81; 95% CI, 0.67-0.99).

**Conclusions and Relevance:**

In this cross-sectional study of surveillance colonoscopy yield in older adults, CRC detection was rare regardless of prior adenoma finding, whereas the advanced neoplasia yield was 12.0% overall. Yields were higher among those with a prior advanced adenoma than among those with prior nonadvanced adenoma and did not increase significantly with age. These findings can help inform whether to continue surveillance colonoscopy in older adults.

## Key Points

**Question:**

What are the colorectal cancer (CRC) and advanced neoplasia yields at surveillance colonoscopy among older patients with a history of colorectal adenoma, and do yields increase with age?

**Findings:**

In this cross-sectional study of 9740 surveillance colonoscopies among 9601 adults aged 70 to 85 years with prior colorectal adenoma, CRC detection at surveillance was 0.3% overall and detection of advanced neoplasia was 12.0%. Yields were higher among patients with a prior advanced adenoma vs nonadvanced adenoma and did not increase significantly with age.

**Meaning:**

In this study, CRC detection at surveillance colonoscopy was rare among older adults regardless of prior adenoma finding, whereas advanced neoplasia detection was more common and more likely in those with a prior advanced adenoma vs nonadvanced adenoma.

## Teaser

This cross-sectional study estimates advanced neoplasia yields during surveillance colonoscopy procedures among adults aged 70 years or older with a history of colorectal adenoma.

## Introduction

Colonoscopy is associated with reduced colorectal cancer (CRC) incidence and mortality through removal of adenomas, the main precursor lesions to CRC, and with decreased mortality through early detection and treatment of cancer.^1,2,3,4,5,6,7,8,9,10,11,12,13,14,15^ Adenomas are found in nearly 40% of screening colonoscopies in the US, and after removal (polypectomy), guidelines recommend that patients undergo future surveillance colonoscopy.^14,15,16,17,18^ However, guidelines provide little direction regarding the age at which colonoscopy surveillance is unlikely to be of substantial benefit and could be stopped.^18,19^ Given the increasing aging population in the US and that nearly 5.6 million adults older than 75 years will undergo surveillance annually by 2024,^20^ estimating the yield of surveillance colonoscopy is important for understanding the balance between potential benefits and known risks of colonoscopy with advancing age.

The risks of colonoscopy increase with age, particularly among those aged 75 years or older, and include heart attack, stroke, sedation-related adverse events (eg, aspiration pneumonia), bleeding, infection, and perforation.^21^ In addition, the benefits of surveillance colonoscopy in older adults may be reduced because of a more limited life expectancy.^19^ Also, in many settings, colonoscopy demand exceeds capacity, and therefore, it is important to direct procedures to those for whom potential benefits will likely outweigh possible harms. These arguments against surveillance colonoscopy in older adults must be weighed against findings that rates of CRC increase with age, at least among unscreened individuals.^22^

In weighing the pros and cons of surveillance colonoscopy in older adults, information needed for shared decision-making between patients and clinicians includes the yields of CRC and advanced neoplasia at surveillance colonoscopy in this age group. Prior studies examining yields in older adults with a history of colorectal polyps have been limited by small sample sizes, limited racial and ethnic representation, and inability to examine yields stratified by prior colonoscopy findings and age.^23,24,25,26,27,28,29^ To address this knowledge gap, we evaluated the surveillance colonoscopy yields of CRC and advanced neoplasia in patients 70 to 85 years of age with a prior adenoma finding from a large, demographically diverse, community-based US health care system. Yields were estimated overall (all ages combined), by age group (70-74, 75-79, and 80-85 years), and by the combination of age group and prior adenoma finding (advanced adenoma vs nonadvanced adenoma).

## Methods

### Study Design

This cross-sectional study evaluated surveillance colonoscopy yields of CRC and advanced neoplasia in patients 70 to 85 years of age with a prior adenoma finding. This study adhered to the Strengthening the Reporting of Observational Studies in Epidemiology (STROBE) reporting guideline for cross-sectional studies. The study was approved by the Kaiser Permanente Northern California (KPNC) institutional review board with a waiver of informed consent because the research involved no more than minimal risk to participants and it could not practically be carried out without the requested waiver.

### Study Setting

Study data were obtained from KPNC, and details of the population and screening practices have been described elsewhere.^30^ The KPNC membership is demographically diverse and similar in socioeconomic characteristics to the region’s diverse census demographics, including the proportions of individuals with commercial insurance, Medicare, and Medicaid.^31^ Thus, studies within this setting approximate community-based research within a demographically diverse population.^32^

### Study Participants

KPNC health plan members were eligible for the study if they were 70 to 85 years of age; underwent a surveillance colonoscopy between January 1, 2017, and December 31, 2019; and had a prior colonoscopy with an adenoma detected (hereafter, “index colonoscopy”) 12 or more months before their surveillance colonoscopy, colonoscopy and pathology reports available for each procedure, and at least 1 year of health plan enrollment prior to the surveillance procedure. Individuals were excluded if, prior to the surveillance colonoscopy, they had a diagnosis of CRC, hereditary CRC syndrome, and/or inflammatory bowel disease; had a prior colectomy; or their surveillance colonoscopy had an inadequate bowel preparation or was not complete to the cecum. The study sample included all patients who met the eligibility criteria.

### Study Outcomes

The outcomes were CRC and advanced neoplasia (either CRC or advanced adenoma). In ascertaining outcome, the most advanced finding from the surveillance procedure was recorded (eg, for a patient diagnosed with both CRC and advanced adenoma, CRC was the recorded finding). Advanced adenoma diagnoses used pathology findings reported at or within 7 days after the procedure. To allow for additional diagnostic procedures for potentially inconclusive examinations, CRC diagnoses were ascertained at or within 180 days after the surveillance colonoscopy.

### Data Sources and Definitions

Data from clinical and administrative databases, including electronic health records, were used to obtain information on cohort member demographic characteristics, diagnoses, pathology findings, and procedures. Race and ethnicity were included in the analysis because some racial and ethnic groups in the US experience inequities in access to and utilization and quality of CRC screening and treatment as well as higher CRC incidence and mortality.^33^ Race and ethnicity data were recorded as 1 of the following 8 categories as documented in the electronic health record: Hispanic; non-Hispanic Alaska Native or American Indian, Asian, Black, Pacific Islander, White, multiracial (reported multiple races), and unknown (race and ethnicity not reported).

Colonoscopies were identified using *Current Procedural Terminology* codes; *International Classification of Diseases, Ninth Revision* and *International Statistical Classification of Diseases and Related Health Problems, Tenth Revision* procedure codes; Healthcare Common Procedure Coding System codes; and site-specific codes. Colonoscopy indication (ie, screening, surveillance, diagnostic, and positive fecal immunochemical test result) was ascertained by a validated colonoscopy indication algorithm based on symptoms and conditions identified using electronic health records.^34,35^ Colonoscopy quality measures (ie, extent of the examination and bowel preparation quality) were ascertained from colonoscopy reports using commercial natural language processing software (Linguamatics I2E; Linguamatics). This approach has been validated in comparison with manual record review.^36^

Adenoma detection and histologic features were ascertained using Systematized Nomenclature of Medicine (SNOMED) coding in electronic pathology databases. Advanced adenoma was defined as a conventional adenoma with high-grade dysplasia or villous or tubulovillous histologic features or as any conventional adenoma 10 mm or greater in size; sessile serrated polyps, traditional serrated polyps, and hyperplastic polyps 10 mm or greater in size were not included in the definition. Nonadvanced adenoma was defined as any conventional adenoma less than 10 mm in size and without high-grade dysplasia or villous or tubulovillous histologic features. Advanced neoplasia was defined as any CRC or advanced adenoma. High-grade dysplasia has no specific SNOMED code and was identified using text string searches of pathology reports. Adenoma size of 10 mm or greater was obtained from a discrete data field within structured colonoscopy flow sheets. Colorectal cancer diagnoses were obtained from the KPNC cancer registry, which reports to the Surveillance, Epidemiology, and End Results program. Colorectal cancer was defined as an adenocarcinoma within the colon or rectum using Surveillance, Epidemiology, and End Results program codes 21040 and 21050; *International Classification of Diseases for Oncology, Third Edition (ICD-O-3)* site (topography) codes C18.0, C18.2-C18.9, C19.9, and C20.9; and *ICD-O-3* histology (morphology) codes 8000, 8010, 8020, 8140, 8143, 8144, 8210, 8211, 8215, 8220, 8221, 8230, 8244, 8245, 8255, 8260-8263, 8323, 8480, 8481, 8490, 8510, 8560, and 8570-8574.

### Statistical Analysis

Summary statistics were used to describe the characteristics of patients who received a surveillance colonoscopy. Surveillance colonoscopy yields were calculated overall (for all ages), by age group (ie, 70-74, 75-79, and 80-85 years), and by both age group and prior adenoma finding (ie, advanced adenoma or nonadvanced adenoma). All surveillance procedures were considered in the yield calculations (ie, patients could contribute >1 procedure to the calculations). Differences in yield measures by age group were assessed using the χ^2^ test of equal proportions. Trends in yields across age groups were evaluated using the Cochran-Armitage test for trend. In the primary analyses, yield measures were calculated using 12 months or longer as the time interval between the surveillance colonoscopy and the index colonoscopy. In sensitivity analyses, yield measures were calculated using 24 or more, 36 or more, 48 or more, and 60 or more months as the time interval to decrease the potential influence of higher-risk patients who may have been recommended to have a relatively early follow-up colonoscopy (ie, within the first few years).

Multivariable logistic regression was used to identify factors associated with advanced neoplasia detection at surveillance, and the odds ratio (OR) with 95% CI was used as an estimate of risk. The variables in the model were patient age (continuous, in years); sex (male or female); race and ethnicity, collapsed into 5 categories (Asian or Pacific Islander, Black, Hispanic, White, and remaining groups [Alaska Native or American Indian, multiracial, and unknown]); body mass index (BMI; calculated as weight in kilograms divided by height in meters squared) of less than 25, 25 to 29.9, or 30 or greater, ascertained at the measurement date closest to the date of the surveillance colonoscopy; tobacco smoking history (ever vs never or unknown); Charlson Comorbidity Index score (0, 1, or ≥2), ascertained in the calendar year before the surveillance colonoscopy; diabetes diagnosis any time prior to the surveillance colonoscopy (yes or no); family history of CRC (yes or no for any relative with CRC); and adenoma findings at the index colonoscopy (advanced or nonadvanced adenoma). In a post hoc analysis, we also included the time interval between the surveillance and index colonoscopies (continuous, in years). Two-sided *P* < .05 indicated statistical significance, and analyses were conducted from September 1, 2022, to February 22, 2024, using SAS, version 9.3 (SAS Institute Inc).

## Results

### Cohort Characteristics

Among 9601 patients 70 to 85 years of age who had an adenoma detected and a follow-up colonoscopy performed 12 or more months after the examination at which the adenoma was detected, 9740 surveillance colonoscopy procedures were performed from 2017 to 2019 (Table 1); 5738 (58.9%) were performed in those aged 70 to 74 years, 3225 (33.1%) in those aged 75 to 79 years, and 777 (8.0%) in those aged 80 to 85. Among the total colonoscopies, 3845 (39.5%) were performed in females and 5895 (60.5%) in males. A total of 29 (0.3%) were in Alaska Native or American Indian patients, 1467 (15.1%) in Asian patients, 523 (5.4%) in Black patients, 899 (9.2%) in Hispanic patients, 28 (0.3%) in Pacific Islander patients, 6711 (68.9%) in White patients, 44 (0.5%) in multiracial patients, and 39 (0.4%) in patients with unknown race and ethnicity. The most prevalent BMI range category was 25 to 29.9 (3951 procedures [40.6%]). Nearly half of procedures were performed among patients who had never smoked tobacco (4864 [49.9%]), 4235 (43.5%) among patients with a Charlson Comorbidity Index score of 2 or higher, 2569 (26.4%) among patients who had diabetes, and 1590 (16.3%) among patients with a documented family history of CRC. The median time interval between the index and surveillance colonoscopies was 4.9 years (IQR, 3.2-5.4 years), and for 2305 (23.7%) of the surveillance colonoscopies, an advanced adenoma had been detected in the index procedure. These 9601 patients comprised the analytic cohort. Baseline characteristics by age group are shown in Table 1.

**Table 1.** Characteristics of Patients Who Underwent a Surveillance Colonoscopy in 2017-2019, Overall and by Age Group

Characteristic^a^	Procedures^b^			
	All (N = 9740)	70-74 y (n = 5738)	75-79 y (n = 3225)	80-85 y (n = 777)
Sex				
Female	3845 (39.5)	2314 (40.3)	1253 (38.9)	278 (35.8)
Male	5895 (60.5)	3424 (59.7)	1972 (61.1)	499 (64.2)
Race and ethnicity				
Hispanic	899 (9.2)	546 (9.5)	285 (8.8)	68 (8.8)
Non-Hispanic				
Alaska Native or American Indian	29 (0.3)	22 (0.4)	7 (0.2)	0
Asian	1467 (15.1)	869 (15.1)	494 (15.3)	104 (13.4)
Black	523 (5.4)	312 (5.4)	167 (5.2)	44 (5.7)
Pacific Islander	28 (0.3)	20 (0.3)	7 (0.2)	1 (0.1)
White	6711 (68.9)	3914 (68.2)	2241 (69.5)	556 (71.6)
Multiracial	44 (0.5)	26 (0.5)	15 (0.5)	3 (0.4)
Unknown	39 (0.4)	29 (0.5)	9 (0.3)	1 (0.1)
BMI				
<25	2917 (29.9)	1628 (28.4)	1015 (31.5)	274 (35.3)
25-29.9	3951 (40.6)	2315 (40.3)	1298 (40.2)	338 (43.5)
≥30	2872 (29.5)	1795 (31.3)	912 (28.3)	165 (21.2)
Tobacco smoking history				
Never smoked or unknown	4864 (49.9)	2933 (51.1)	1543 (47.8)	388 (49.9)
Ever smoked	4876 (50.1)	2805 (48.9)	1682 (52.2)	389 (50.1)
Charlson Comorbidity Index score				
0	2978 (30.6)	1982 (34.5)	829 (25.7)	167 (21.5)
1	2527 (25.9)	1454 (25.3)	855 (26.5)	218 (28.1)
≥2	4235 (43.5)	2302 (40.1)	1541 (47.8)	392 (50.5)
Family history of colorectal cancer (any relative)	1590 (16.3)	968 (16.9)	496 (15.4)	126 (16.2)
Diabetes diagnosis	2569 (26.4)	1509 (26.3)	878 (27.2)	182 (23.4)
Time interval between index and surveillance colonoscopies, median (IQR), y				
All	4.9 (3.2-5.4)	4.9 (3.2-5.4)	4.7 (3.2-5.4)	4.3 (3.2-5.4)
Group with nonadvanced adenoma	5.1 (3.4-5.5)	5.1 (3.5-5.5)	5.0 (3.4-5.5)	5.0 (3.6-5.6)
Group with advanced adenoma	3.3 (3.1-4.3)	3.3 (3.0-4.3)	3.3 (3.0-4.2)	3.3 (3.0-4.3)
Adenoma at index colonoscopy				
Nonadvanced	7435 (76.3)	4389 (76.5)	2466 (76.5)	580 (74.6)
Advanced	2305 (23.7)	1349 (23.5)	759 (23.5)	197 (25.4)

Abbreviation: BMI, body mass index (calculated as weight in kilograms divided by height in meters squared).

^a^ All covariates were ascertained at the time of the surveillance colonoscopy except weight for the BMI calculation (obtained at the measurement date closest to the date of the index colonoscopy) and Charlson Comorbidity Index score (ascertained in the calendar quarter before the surveillance colonoscopy).

^b^ Data are presented as number (percentage) of procedures unless otherwise indicated.

### Surveillance Colonoscopy Yields Overall and by Age Group

Among the 9740 surveillance colonoscopies, CRC was the most advanced finding for 28 (0.3%), advanced adenoma for 1141 (11.7%), and advanced neoplasia (CRC or advanced adenoma) for 1169 (12.0%) (Figure and Table 2). Surveillance colonoscopy yields did not increase significantly with age (Figure and Table 2). Among those aged 70 to 74, 75 to 79, and 80 to 85 years, CRC yields were 0.2% (12 of 5738), 0.4% (13 of 3225), and 0.4% (3 of 777), respectively (test for trend, *P* = .12). Advanced adenoma yields were 11.8% (679 of 5738), 11.3% (364 of 3225), and 12.6% (98 of 777), respectively (test for trend, *P* = .99). Advanced neoplasia yields were 12.0% (691 of 5738), 11.7% (377 of 3225), and 13.0% (101 of 777), respectively (test for trend, *P* = .79). In sensitivity analyses, yield estimates did not differ substantially when the time interval between the index and surveillance colonoscopies was restricted to 24 or more, 36 or more, 48 or more, and 60 or more months with the exception that when restricted to 60 or more months, the increase in CRC yield with age was significant (Table 2).

**Figure.**
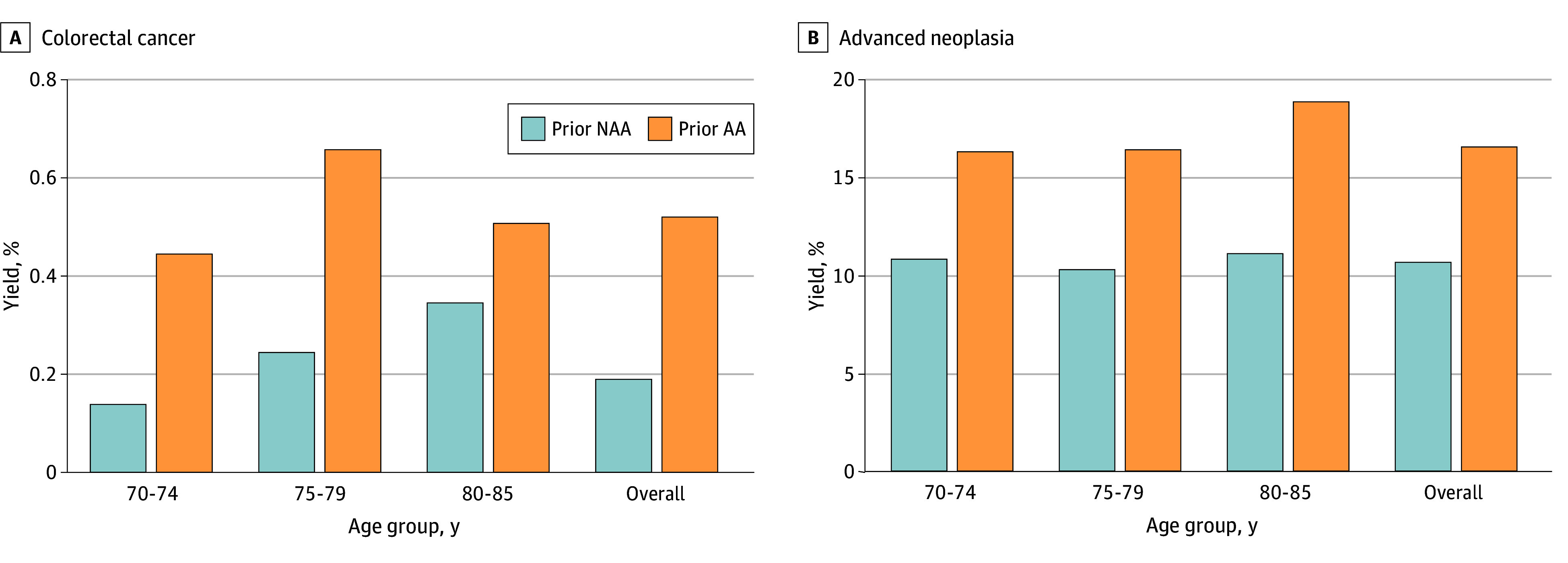
Colorectal Cancer and Advanced Neoplasia Yields at Surveillance Colonoscopy by Age Group and Prior Adenoma Findings Yields were calculated using 12 or more months as the time interval between the surveillance colonoscopy and the index colonoscopy. AA indicates advanced adenoma; NAA, nonadvanced adenoma.

**Table 2.** Surveillance Colonoscopy Neoplasia Yields Overall and by Age Group

Interval, finding^a^	Yield, No./total No. of procedures (%)				Trend *P* value^b^
	All	Age 70-74 y	Age 75-79 y	Age 80-85 y	
≥12-mo Interval					
Colorectal cancer	28/9740 (0.3)	12/5738 (0.2)	13/3225 (0.4)	3/777 (0.4)	.12
Advanced adenoma	1141/9740 (11.7)	679/5738 (11.8)	364/3225 (11.3)	98/777 (12.6)	.99
Advanced neoplasia	1169/9740 (12.0)	691/5738 (12.0)	377/3225 (11.7)	101/777 (13.0)	.79
≥24-mo Interval					
Colorectal cancer	27/9456 (0.3)	11/5562 (0.2)	13/3135 (0.4)	3/759 (0.4)	.09
Advanced adenoma	1104/9456 (11.7)	658/5562 (11.8)	350/3135 (11.2)	96/759 (12.6)	.95
Advanced neoplasia	1131/9456 (12.0)	669/5562 (12.0)	363/3135 (11.6)	99/759 (13.0)	.83
≥36-mo Interval					
Colorectal cancer	26/9056 (0.3)	10/5319 (0.2)	13/3010 (0.4)	3/727 (0.4)	.06
Advanced adenoma	1056/9056 (11.7)	635/5319 (11.9)	329/3010 (10.9)	92/727 (12.7)	.70
Advanced neoplasia	1082/9056 (11.9)	645/5319 (12.1)	342/3010 (11.4)	95/727 (13.1)	.95
≥48-mo Interval					
Colorectal cancer	23/6866 (0.3)	9/3984 (0.2)	11/2294 (0.5)	3/588 (0.5)	.08
Advanced adenoma	788/6866 (11.5)	460/3984 (11.5)	259/2294 (11.3)	69/588 (11.7)	.94
Advanced neoplasia	811/6866 (11.8)	469/3984 (11.8)	270/2294 (11.8)	72/588 (12.2)	.81
≥60-mo Interval					
Colorectal cancer	20/5873 (0.3)	7/3413 (0.2)	10/1964 (0.5)	3/496 (0.6)	.04
Advanced adenoma	659/5873 (11.2)	378/3413 (11.1)	225/1964 (11.5)	56/496 (11.3)	.73
Advanced neoplasia	679/5873 (11.6)	385/3413 (11.3)	235/1964 (12.0)	59/496 (11.9)	.48

^a^ Intervals were calculated as the time between the surveillance and the index colonoscopies.

^b^ *P* values for the Cochran-Armitage test for trend across age groups.

### Surveillance Colonoscopy Yields Overall and by Age Group Stratified by Prior Adenoma Finding

At an interval of 12 or more months between colonoscopies, patients with a prior advanced adenoma had significantly higher yields of advanced neoplasia (380 of 2305 [16.5%] vs 789 of 7435 [10.6%]; *P* < .001) and CRC (12 of 2305 [0.5%] vs 16 of 7435 [0.2%]; *P* = .02) compared with those with a prior nonadvanced adenoma (Figure and Table 3). The advanced neoplasia yields at the surveillance colonoscopy did not differ with increasing age for those with prior advanced adenoma (70-74 years: 219 of 1349 [16.2%]; 75-79 years: 124 of 759 [16.3%]; 80-85 years: 37 of 197 [18.8%]; test for trend, *P* = .49) or nonadvanced adenoma (70-74 years: 472 of 4389 [10.8%]; 75-79 years: 253 of 2466 [10.3%]; 80-85 years: 64 of 580 [11.0%]; test for trend, *P* = .82) (Figure and Table 3). Similarly, CRC yields did not differ with increasing age for those with a prior advanced adenoma (70-74 years: 6 of 1349 [0.4%]; 75-79 years: 5 of 759 [0.7%]; 80-85 years: 1 of 197 [0.5%]; test for trend *P* = .66) or nonadvanced adenoma (70-74 years: 6 of 4389 [0.1%]; 75-79 years: 8 of 2466 [0.3%]; 80-85 years: 2 of 580 [0.3%]; test for trend, *P* = .10) (Table 3). In sensitivity analyses, in surveillance colonoscopies both among patients with a prior advanced adenoma and patients with a prior nonadvanced adenoma, yield estimates did not differ substantially when the time interval between the surveillance colonoscopy and the index colonoscopy was restricted to 24 or more, 36 or more, 48 or more, and 60 or more months (Table 3).

**Table 3.** Colorectal Cancer and Advanced Neoplasia Yield at Surveillance Colonoscopy by Prior Adenoma Finding Overall and by Age Group

Interval, prior adenoma finding^a^	Overall		Age 70-74 y		Age 75-79 y		Age 80-85 y		Trend *P* value^c^
	No./total No. (%)	*P* value^b^	No./total No. (%)	*P* value^b^	No./total No. (%)	*P* value^b^	No./total No. (%)	*P* value^b^	
**≥12-mo Interval**									
Colorectal cancer yield									
NAA	16/7435 (0.2)	.02	6/4389 (0.1)	.03	8/2466 (0.3)	.20	2/580 (0.3)	.75	.10
AA	12/2305 (0.5)		6/1349 (0.4)		5/759 (0.7)		1/197 (0.5)		.66
Advanced neoplasia yield									
NAA	789/7435 (10.6)	<.001	472/4389 (10.8)	<.001	253/2466 (10.3)	<.001	64/580 (11.0)	.005	.82
AA	380/2305 (16.5)		219/1349 (16.2)		124/759 (16.3)		37/197 (18.8)		.49
**≥24-mo Interval**									
Colorectal cancer yield									
NAA	17/7319 (0.2)	.07	6/4313 (0.1)	.07	9/2433 (0.4)	.47	2/573 (0.3)	.72	.07
AA	10/2137 (0.5)		5/1249 (0.4)		4/702 (0.6)		1/186 (0.5)		.64
Advanced neoplasia yield									
NAA	786/7319 (10.7)	<.001	474/4313 (11.0)	<.001	248/2433 (10.2)	<.001	64/573 (11.2)	.007	.62
AA	345/2137 (16.1)		195/1249 (15.6)		115/702 (16.4)		35/186 (18.8)		.29
**≥36-mo Interval**									
Colorectal cancer yield									
NAA	17/7107 (0.2)	.10	6 /4183 (0.1)	.15	9/2365 (0.4)	.41	2/559 (0.4)	.67	.08
AA	9/1949 (0.5)		4/1136 (0.4)		4/645 (0.6)		1/168 (0.6)		.45
Advanced neoplasia yield									
NAA	779/7107 (11.0)	<.001	468/4183 (11.2)	<.001	245/2365 (10.4)	<.001	66/559 (11.8)	.07	.78
AA	303/1949 (15.5)		177/1136 (15.6)		97/645 (15.0)		29/168 (17.3)		.81
**≥48-mo Interval**									
Colorectal cancer yield									
NAA	17/5756 (0.3)	.20	6/3377 (0.2)	.13	9/1915 (0.5)	.88	2/464 (0.4)	.60	.08
AA	6/1110 (0.5)		3/607 (0.5)		2/379 (0.5)		1/124 (0.8)		.72
Advanced neoplasia yield									
NAA	640/5756 (11.1)	<.001	374/3377 (11.1)	.001	217/1915 (11.3)	.14	49/464 (10.6)	.02	.94
AA	171/1110 (15.4)		95/607 (15.7)		53/379 (14.0)		23/124 (18.5)		.77
**≥60-mo Interval**									
Colorectal cancer yield									
NAA	16/5043 (0.3)	.45	6/2960 (0.2)	.94	8/1681 (0.5)	.61	2/402 (0.5)	.52	.11
AA	4/830 (0.5)		1/453 (0.2)		2/283 (0.7)		1/94 (1.1)		.21
Advanced neoplasia yield									
NAA	558/5043 (11.1)	.003	320/2960 (10.8)	.03	199/1681 (11.8)	.67	39/402 (9.7)	.002	.89
AA	121/830 (14.6)		65/453 (14.3)		36/283 (12.7)		20/94 (21.3)		.29

Abbreviations: AA, advanced adenoma; NAA, nonadvanced adenoma.

^a^ Intervals were calculated as the time between the surveillance and the index colonoscopies.

^b^ *P* values for the comparison of yield proportions in patients with a prior NAA vs AA by χ^2^ test of equal proportions.

^c^ *P* values for the χ^2^ test for trend across age groups.

### Factors Associated With Advanced Neoplasia Detection at Surveillance Colonoscopy

In a multivariable analysis (Table 4), factors associated with the detection of advanced neoplasia at surveillance colonoscopy were prior advanced adenoma (adjusted OR [AOR], 1.65; 95% CI, 1.44-1.88), BMI of 30 or greater vs less than 25 (AOR, 1.21; 95% CI, 1.03-1.44), and having ever smoked tobacco (AOR, 1.14; 95% CI, 1.01-1.30). Compared with White patients, Asian or Pacific Islander patients were less likely to have advanced neoplasia detected at surveillance colonoscopy (AOR, 0.81; 95% CI, 0.67-0.99). In a post hoc analysis, the time interval between colonoscopies was not associated with advanced neoplasia detection and did not affect risk estimates for other factors.

**Table 4.** Factors Associated With Advanced Neoplasia Detection at Surveillance Colonoscopy

Factor	Adjusted odds ratio (95% CI)^a^
Age, y	
70-74	1 [Reference]
75-79	0.96 (0.84-1.10)
80-85	1.09 (0.87-1.37)
Sex	
Female	1 [Reference]
Male	1.03 (0.91-1.18)
Race and ethnicity	
Hispanic	0.86 (0.68-1.07)
Non-Hispanic Asian or Pacific Islander	0.81 (0.67-0.99)
Non-Hispanic Black	1.00 (0.76-1.30)
Non-Hispanic White	1 [Reference]
Remaining groups^b^	1.09 (0.63-1.89)
BMI	
<25	1 [Reference]
25-29.9	1.05 (0.90-1.23)
≥30	1.21 (1.03-1.44)
Tobacco smoking history	
Never smoked or unknown	1 [Reference]
Ever smoked	1.14 (1.01-1.30)
Charlson Comorbidity Index score	
0	1 [Reference]
1	1.07 (0.90-1.26)
≥2	1.07 (0.91-1.27)
Diabetes diagnosis	
No	1 [Reference]
Yes	1.06 (0.90-1.24)
Family history of colorectal cancer	
No	1 [Reference]
Yes	1.01 (0.86-1.20)
Prior advanced adenoma	
No	1 [Reference]
Yes	1.65 (1.44-1.88)

Abbreviation: BMI, body mass index (calculated as weight in kilograms divided by height in meters squared).

^a^ Adjusted for patient age, sex, race and ethnicity, BMI, tobacco smoking status, Charlson Comorbidity Index score, family history of colorectal cancer, and index colonoscopy finding. All covariates were ascertained at the time of the surveillance colonoscopy except weight and height for the BMI calculation (obtained at the measurement date closest to the date of the index colonoscopy) and Charlson Comorbidity Index score (ascertained in the calendar year before the surveillance colonoscopy).

^b^ Includes non-Hispanic Alaska Native or American Indian, multiracial (reported as multiple races), and unknown (race and ethnicity not reported) as documented in electronic health records.

## Discussion

In a large, integrated health care system, among 9740 surveillance colonoscopies in patients 70 to 85 years of age with a history of colorectal adenoma, detection of CRC or advanced neoplasia did not increase significantly with age. Overall, CRC detection was rare (0.3%), while detection of advanced neoplasia was more common (12.0%). Patients with a history of advanced adenoma vs nonadvanced adenoma were more likely to have CRC detected, though still rarely (0.5% vs 0.2%), and were more likely to have advanced neoplasia detected (16.5% vs 10.6%). These findings provide some of the first large-scale, community-based information on the yield of surveillance colonoscopy among older adults.

Prior studies of surveillance colonoscopy yield in older populations have reported conflicting results. Four studies reported that the prevalence of CRC and adenoma detection increased with age.^23,24,25,26^ For example, a study using the New Hampshire Colonoscopy Registry showed that CRC yield at surveillance colonoscopy was low but increased from 0.4% to 0.6% and 0.8% among older adults aged 70-74, 75-79, and 80-84 years, respectively.^23^ In contrast, 3 other studies reported that CRC prevalence decreased with age.^27,28,29^ The inconsistency in prior studies may stem from the wide range of age groups studied (ie, 50-100 years of age), date of publication (given the improvement in colonoscopy techniques and adenoma detection in the past 10-15 years), different cohort sizes (ie, 80-42 611), and varying colonoscopy indications (ie, diagnostic, screening, and/or surveillance) in the study samples. We included only patients 70 to 85 years of age undergoing a surveillance colonoscopy following a colonoscopy in which adenomatous polyps were removed, and the yield estimates represent a contemporary population with regard to colonoscopy quality. Our surveillance colonoscopy yield estimates among older adults with a history of polyps are similar to or slightly higher than rates reported in the literature among individuals younger than 70 years, as would be expected given the older population in our study.^37^ Based on a recent systematic review and meta-analysis, the yield of CRC among patients aged 50 to 70 years undergoing surveillance for a history of polyps ranged from 0.5% to 2.3%, with a pooled prevalence or yield of 1.4%.^37^ In addition, yield of advanced polyps among patients aged 50 to 70 years undergoing surveillance for a history of polyps ranged from 2.9% to 24.4%, with a pooled prevalence or yield of 8.2%.^37^

Current US guidelines do not provide a recommendation for the age to stop surveillance but advocate for the decision to be individualized based on benefits, risks, patient health status, and patient preferences.^17,18^ The current study provides 2 key findings that can inform shared decision-making between patients and their clinicians. First, CRC detection at surveillance colonoscopy was rare among older adult patients with prior advanced or nonadvanced adenomas. Thus, for many older adults, particularly those with a prior nonadvanced adenoma, the low rate of CRC detection at surveillance may not justify the potential harms and burdens of colonoscopy that may increase with age. However, for some older adults with a predicted life expectancy of 10 or more years and without significant competing comorbidities, especially for those with a prior advanced adenoma, detection of early-stage CRC or advanced adenomas at surveillance could lead to earlier treatment and improved outcomes. Second, advanced adenoma detection at surveillance colonoscopy, which also did not increase with age, was more common than CRC detection; however, advanced adenomas themselves are not harmful to patients, and for the rare lesions that do progress to invasive cancer, the process takes several years.^38^ Thus, among older adults with limited life expectancies due to comorbidities, few would likely benefit from the detection and removal of these polyps. The current data can help to estimate potential yields and benefits that can be considered vs the risks of sedation (eg, aspiration) and other potential colonoscopy-related harms (eg, perforation, major gastrointestinal bleeding), which increase with age.^21,39,40,41^

### Strengths and Limitations

Strengths of the study include the use of data from a large, demographically diverse, community-based health care system, which allowed access to comprehensive information about colonoscopy indications and findings. In addition, the large study size allowed for a specific focus on patients aged 70 years or older and stratifications by prior adenoma findings to provide patients and clinicians with granular data to inform individual decision-making regarding which older patients may be most likely to benefit from continued colonoscopy surveillance and which could potentially stop surveillance.

The study also had several limitations that should be considered. First, the study population was from a large, integrated health care system and limited to patients with a prior adenoma who had a complete colonoscopy with adequate bowel preparation; thus, the results may not be generalizable to unscreened populations or those with incomplete screening (who might be expected to be at higher risk). Second, the colonoscopy indication algorithm used may have misclassified some procedures as surveillance rather than diagnostic; however, validation studies have shown that it has high specificity (ie, 95%-96%) for detecting surveillance colonoscopies.^34,35^ Third, our advanced adenoma definition did not include traditional serrated polyps or sessile serrated polyps given these do not have formal SNOMED codes; however, contemporary patients with such polyps have natural histories similar to those of patients with similarly sized polyps classified as traditional adenomas, particularly for small, serrated polyps. Fourth, family history data were collected through different data sources and aggregated in this analysis as any family history of CRC regardless of the degree of the relative, which may dilute its effect as a risk factor for CRC given that patients with a family history may have a greater risk of CRC. Fifth, caution should be used in drawing conclusions from our findings since the study was cross-sectional, the follow-up time was limited, and CRC development may take many years.

## Conclusions

In this cross-sectional study, overall, CRC detection was rare and the yield of advanced neoplasia at surveillance colonoscopy was 12.0% among older adults in a large, community-based setting. Yields were higher in those with a prior advanced adenoma vs nonadvanced adenoma, and yields did not increase significantly with age. With current guidelines offering no specific age at which to stop surveillance colonoscopy, the study findings can inform clinicians and older patients regarding the potential benefits (or lack of benefits) of continuing with postpolypectomy surveillance in the context of the life expectancy of the patient and weighed against the rare but known harms of colonoscopy, which increase with advancing age and comorbidities.

## Notes

Supplement.
Data Sharing Statement
