# Supplementary material for: Surveillance Colonoscopy Findings in Older Adults With a History of Colorectal Adenomas
Source: JAMA Netw Open. 2024 Apr 2;7(4):e244611. doi: 10.1001/jamanetworkopen.2024.4611 (PMC10988351; doi:10.1001/jamanetworkopen.2024.4611)
Supplement: Supplement. — Data Sharing Statement [file jamanetwopen-e244611-s001.pdf]

Data Sharing Statement

Lee. Surveillance Colonoscopy Findings in Older Adults With a History of Colorectal Adenomas. *JAMA Netw Open*. Published April 02, 2024.
doi:10.1001/jamanetworkopen.2024.4611

Data
Data available: No
